# Evaluation of temperature induction in focal ischemic thermocoagulation model

**DOI:** 10.1371/journal.pone.0200135

**Published:** 2018-07-05

**Authors:** Helio da Silva, Mariana P. Nucci, Javier B. Mamani, Rosalia Mendez-Otero, Leopoldo P. Nucci, Alberto Tannus, Lionel F. Gamarra

**Affiliations:** 1 Hospital Israelita Albert Einstein, São Paulo, Brazil; 2 Santa Casa Misericórdia de São Paulo, São Paulo, Brazil; 3 Hospital das Clínicas HCFMUSP, Universidade de São Paulo, São Paulo, SP–Brazil; 4 Instituto de Biofísica Carlos Chagas Filho, Universidade Federal do Rio de Janeiro, Rio de Janeiro, Brazil; 5 Universidade Federal de São Paulo, São Paulo, Brazil; 6 CIERMag-Instituto de Física de São Carlos, Universidade de São Paulo, São Paulo, Brazil; Fraunhofer Research Institution of Marine Biotechnology, GERMANY

## Abstract

The thermocoagulation model, which consists of focal cerebral ischemia with craniectomy, is helpful in studying permanent ischemic brain lesions and has good reproducibility and low mortality. This study analyzed the best conditions for inducing a focal ischemic lesion by thermocoagulation. We investigated parameters such as temperature and thermal dissipation in the brain tissue during induction and analyzed real-time blood perfusion, histological changes, magnetic resonance imaging (MRI), and motor behavior in a permanent ischemic stroke model. We used three-month-old male Wistar rats, weighing 300–350 g. In the first experiment, the animals were divided into four groups (*n* = 5 each): one sham surgery group and three ischemic lesion groups having thermocoagulation induction (TCI) temperatures of 200°C, 300°C, and 400°C, respectively, with blood perfusion (basal and 30 min after TCI) and 2,3,5-Triphenyl-tetrazolium chloride (TTC) evaluation at 2 h after TCI. In the second experiment, five groups (*n* = 5 each) were analyzed by MRI (basal and 24 h after TCI) and behavioral tests (basal and seven days after TCI) with the control group added for the surgical effects. The MRI and TTC analyses revealed that ischemic brain lesions expressively evolved, especially at TCI temperatures of 300°C and 400°C, and significant motor deficits were observed as the animals showed a decrease frequency of movement and an asymmetric pattern. We conclude that a TCI temperature of 400°C causes permanent ischemic stroke and motor deficit.

## Introduction

Stroke, a cerebrovascular disease (CVD), is the main cause of temporary or permanent neurologic disabilities in individuals aged 50 years and older. According to the World Health Organization, CVD was the second leading cause of death in the world in 2014 behind ischemic heart disease [1].

The origin of stroke is multifactorial, and vessel bruit due to hypertension is the major risk [2]. The use of animal models in recent years has provided a better understanding of the pathophysiologic mechanisms of strokes and allowed preclinical tests of new therapeutic agents. However, results obtained in animal models have not been widely applied in treating stroke patients. Most animal models for ischemic stroke target the middle cerebral artery (MCA) region to mimic the more frequent clinical situation, and currently used animal models produce either a permanent or transient ischemia [3]. Recent reviews of preclinical models of stroke [4,5,6] have shown that cell death and survival mechanisms as well as neuronal plasticity should be investigated after controlling for some confounders of the transition to the clinic. Although findings from preclinical research have improved comprehension of the ischemic process, some clinical conditions remain as challenges yet to be elucidated [7].

The thermocoagulation model is a type of focal cerebral ischemia with craniectomy. It represents an opportunity to study permanent ischemic brain lesions with good reproducibility and low mortality [5]. In this model, the pial vessels are cauterized followed by heat exchange in the brain tissue with a hot probe in specific regions of the cerebral cortex. The tissue and vascular changes mimic ischemic damage in humans due to bioenergetic insufficiency from decreased oxygen and glucose as several neurochemical processes are suppressed and ATP production is restricted. The decrease in intracellular ATP rapidly restricts energy-dependent ion transport relating to Na^+^ / K^+^ ATPase, resulting in depolarization of neurons and glia [8,9], activation of voltage-dependent calcium channels, and the release of excitatory amino acids such as glutamate in the extracellular space [10].

Given the model’s relevance for stroke studies, important aspects such as the ideal temperature for thermocoagulation induction (TCI), duration of induction to obtain a complete lesion, and the adequate blood perfusion rate in the ischemic area remain unclear [11,12–18]. Thus, this study seeks to establish the most suitable conditions for induction of an ischemic lesion. Temperature of induction and thermal dissipation in the brain tissue were investigated through real-time blood perfusion, histological analysis, magnetic resonance imaging (MRI), and behavior assessment to develop an adequate model of the permanent focal ischemic stroke.

## Materials and methods

### Animals and experimental groups

We used three-month-old male Wistar rats weighing 300–350 g. The animals were housed (two rats per cage) for a week’s acclimation and quarantine. Throughout the experiments, the animals were at the vivarium of the Experimental Surgical Training Center (Centro de Experimentação e Treinamento em Cirurgia–CETEC), maintained at 21 ± 2°C and 60 ± 5% relative humidity with full ventilation and a 12 h light/dark cycle (7 am to 7 pm). They had access to food and water *ad libitum*. The vivarium is accredited by the Association for the Assessment and Accreditation of Laboratory Animal Care International (AAALAC International), and the general conditions were monitored daily. The Ethics in Animal Research Committee of the Hospital Israelita Albert Einstein (HIAE) had approved this study with approval number 683–09.

### Experimental design

**Fig 1:**
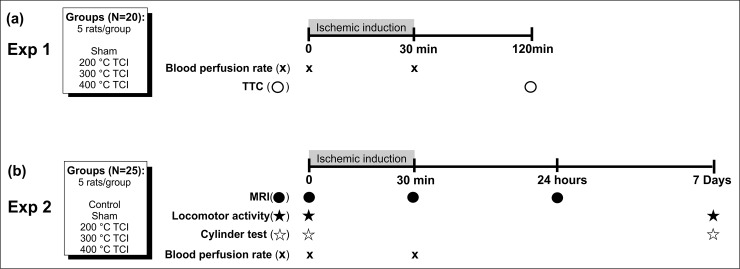
Experimental design, animal groups classification, and timeline. (a) Experiment 1: Four groups of animals (five rats per group) were used. Blood perfusion rate was evaluated at basal time (after craniectomy) and 30 min after ischemic induction, and the TTC analysis was performed 120 min after ischemic induction. (b) Experiment 2: Five groups of animals (five rats per group) were used. MRI, locomotor activity, and cylinder test were evaluated at basal time (before surgery for ischemic induction) while the blood perfusion rate was measured after craniectomy (basal time). MRI and blood perfusion rate were evaluated again at 30 min after ischemic induction, and a third MRI was performed 24 h after induction. Locomotor activity analysis and cylinder test were repeated seven days after the ischemic induction. The timeline in the figure shows the sequence of the experiments.

Two separate experiments were performed (Fig 1, Experiments 1 and 2).

*Experiment 1 –*Four groups (*n* = 5 each) were formed to evaluate the TCI model: sham surgery and three ischemic lesion groups with TCI temperatures of 200°C, 300°C, and 400°C, respectively. Blood perfusion rate was evaluated by Perican at basal time (after craniectomy) and after 30 min of TCI as also infarct analysis by 2,3,5-Triphenyl-tetrazolium chloride (TTC) 120 min after TCI.

*Experiment 2 –*The TCI model was evaluated for structural and behavioral brain function in five groups (*n* = 5 each): control, sham surgery, and three ischemic lesion groups with TCI temperatures of 200°C, 300°C, and 400°C, respectively. Three measures of MRI and blood perfusion rate and two rounds of behavioral tests were performed. MRI was obtained at basal time (before the surgery), after 30 min of TCI, and after 24 h. Blood perfusion rate was analyzed after craniectomy and following the second and third measures of MRI. The first behavioral test was conducted before the surgery and the second seven days after TCI.

The animals were randomly allocated to nine groups (four groups for the first experiment and five groups for the second), coded, and housed in individual cages. One investigator performed the first set of experiments (ischemic induction, blood perfusion rate, and TTC analysis) and the ischemic induction of the animals of the second experiment, while another blinded investigator carried out the MRI and behavioral tests.

### Thermocoagulation induction in somatosensory cortex vessels

A focal ischemic lesion was induced by thermocoagulation of blood in the pial blood vessels of primary motor and somatosensory cortex, as previously described [11–22]. Briefly, the animals were anesthetized with ketamine hydrochloride (90 mg/kg, i.p.) and xylazine hydrochloride (12 mg/kg, i.p.) and placed in a stereotaxic apparatus (Harvard Apparatus, Holliston, United States). A craniectomy was made to expose the left somatosensory cortex, region of the left middle cerebral artery (MCA) (+ 2 mm to -6 mm in anterior-posterior (AP) and +2 mm in medial-lateral (ML) axis from Bregma), according to the atlas of Paxinos and Watson [23]. Basal blood perfusion image was acquired in the exposed brain area with a PeriCam Perfusion Speckle Imager (PSI) system (Perimed, Stockholm, Sweden) before induction. Thermocoagulation was performed in three different animal groups at temperatures of 200°C, 300°C, or 400°C, respectively, providing a better dynamic evaluation of the lesion in the pial blood vessels of the somatosensory cortex according to the temperature used. Superficial blood vessels of the left sensorimotor brain area were thermocoagulated transdurally by a hot probe into the dura matter (~2 mm) for 30 minutes. The hot probe (ESD-900 model, Instrutherm, Brazil) had digital control for temperature (150°C–480°C), 50 W, and ± 1°C of accuracy and maintained constant temperature. After 30 min of TCI at 400°C, vessel lesions were evaluated macroscopically through the color changes in the region targeted, from light red to dark red to indicate complete thermocoagulation of blood [11–15,19–22]. Thus TCI was performed for 30 min at the three temperatures. A decrease in blood perfusion by 75% of the initial measure (before induction) after 30 min of TCI was considered as a parameter for complete ischemic lesion [24, 25]. The procedure was completed with suturing of the incision tissue and topical application of lidocaine and IV administration of tramadol (5 mg/kg) every 12 h for six days. Throughout anesthesia, the rats were placed on a heating pad to maintain the rectal temperature at 37.0°C ± 0.5°C (PhysioSuite, kent Scientific Corporation, Torrington, CT, USA). Animals from the sham group underwent the whole procedure up to, but not including the TCI. The probe was positioned turned off for 30 min and thereafter, the incision tissue was sutured. This protocol was registered in the Protocols.io site that provided the following DOI: dx.doi.org/10.17504/protocols.io.pasdiee [PROTOCOL DOI]

### Evaluation of thermal dissipation in brain tissue by hot probe

The changes in brain tissue temperature induced by the probe were measured spatially and directly on the tissue. Spatially, the temperature was measured using an infrared camera CA1886 (RAYCAM, 9Hz, CHAUVIN®), with emissivity of 0.95, distance of 30 cm, temperature accuracy ± 1°C, and resolution 160 x 120 and directly on the brain tissue by using Fiber Optic Thermometer Systems (FOB100, Omega, USA), with 0.1°C of resolution and ± 1°C of accuracy through the Fiber Optic Temperature Probe (T1C-01-B05, Qualitrol, USA).

### Real-time blood perfusion imaging analysis of an ischemic lesion

Blood perfusion rate was evaluated in the ischemic lesional area in all animals after craniectomy with a PeriCam PSI System (Perimed, Stockholm, Sweden) for analysis of complete occlusion after stroke induction [26,27]. This system provides images using Laser Speckle Contrast Analysis (LASCA) technology, and data on both the dynamics and the spatial distribution of the perfusion throughout the procedure are displayed in real time. During the procedure, environmental temperature was controlled to approximate 23°C ± 1°C and the relative humidity between 50% and 60% whereas the evaluated field was not exposed to direct light. The PSI parameter was set as follows: image acquisition rate, 50 Hz; normal resolution, 0.5 mm; 1 frame per second; 10 ± 1 cm of working distance; 10 (height) x 8 (width) cm^2^ of monitor area and 1.0 cm^2^ of region of interest (ROI). PIMSoft v 1.54 (Perimed, Stockholm, Sweden) was used for recording, saving, and analysis of data. Perfusion unit (PU) was the unit used and the higher the PU, the greater the perfusion observed.

### Magnetic resonance imaging (MRI)

MRI was acquired at basal time, at 30 min, and 24 h after stroke induction by thermocoagulation.

MRI was performed on a 2T/30 cm bore superconducting magnet 85310HR model (Oxford Instruments, Abingdon, UK), interfaced to a Bruker Avance AVIII console (Bruker-Biospin, Inc., Billerica, MA, U.S.A) with Paravision 5.0. Software (Bruker-Biospin, Inc., Billerica, MA, U.S.A). A crossed saddle radiofrequency coil [28] was used as a head probe in rats anesthetized with an IP injection of ketamine hydrochloride (90 mg/kg) and xylazine hydrochloride (12 mg/kg).

T2-weighted images were acquired by using a rapid acquisition with relaxation enhancement sequence (RARE), with repetition time (TR)/echo time (TE) 5000/51.0 ms, rare factor = 6, 20 slices with 0.5 mm of thickness without gap, FOV = 15 x 30 mm^2^, matrix 96 x 96, spatial resolution 208 x 208, 24 averages and frequency of 12.5 kHz for morphometric analysis. Total experimental time per animal was 20 minutes.

Detectable ischemic lesion slices were selected from the 20 slices acquired in morphometric analysis. In these slices, the region of interest (ROI) was delineated on the ischemic lesion by using the ImageJ software [29], and the ischemic lesion volume was calculated as the sum of lesion areas in each slice multiplied by the thickness of the slice.

### TTC staining of ischemic lesion

At 2 h post ischemic induction, the animals were fully anesthetized with an overdose of ketamine hydrochloride (90 mg/kg, IP) and xylazine hydrochloride (12 mg/kg, IP) and decapitated. Brains were removed and cut at 2 mm. The brain slices were fixed in a petri dish, immediately immersed in a solution of 2,3,5-Triphenyl-tetrazolium chloride (TTC, Sigma-Aldrich, USA) at 40°C for 30 min, and transferred in 4% paraformaldehyde (PFA) at 4°C. TTC staining (red) represents normal brain tissue, and infarcted area is stained pale (white) or unstained.

### Behavior assessment in ischemic model

Animals were assessed for motor, gait, and spontaneous movements before stroke induction (basal—day 0) and at seven days post stroke. Testing was carried out at the same time of the day for each session to limit variability in circadian activity.

### Spontaneous locomotor activity (Actimeter)

The spontaneous global locomotor activity was measured by the Infrared (IR) Actimeter LE 8825 systems (Actitrack, Panlab Harvard Apparatus, Barcelona, Spain). The apparatus consists of a two-dimensional (X and Y axes) square frame of 450 x 450 mm^2^, surrounded by transparent walls 30 cm high, a frame support, and a control unit. Each frame counts 16 x 16 infrared beams for optimal subject detection used for evaluation of general activity, locomotor and stereotyped movements, or rearing or exploration (nose-spoke detection in the hole-board option). Briefly, global locomotor activity was quantified by using activity cages equipped with two horizontal infrared beams located one above another 4 cm and 8 cm above the cage floor. Each animal was placed in the center of the arena, and its spontaneous behavior was tracked for 5 min.

During the test, horizontal locomotor activity (movements) was determined by breaks in movement-sensitive photobeams that were then converted into locomotor activity counts, and vertical activity was recorded as the number of rearing episodes breaking the photocell beams of the upper frame. The threshold for the upper and lower frames were 10 s and 5 s, respectively to determine the speed of movement (slow or fast). The six parameters used for comparison between groups and sessions were slow movements (S-MOV), fast movements (F-MOV), slow stereotyped (S-STE), fast stereotyped (F-STE) slow rearing (S-REA), and fast rearing (F-REA). The data were processed using SEDACOM software v2.0.

At the end of the session, the animals were returned to their home cage. The arena and walkway were wiped with 5% alcohol to avoid olfactory cues.

### Analysis of forelimb function (cylinder test)

The cylinder sensorimotor test provides a way to evaluate a rodent´s spontaneous use of forelimbs and has been used in many stroke models [30]. To evaluate forelimb deficits, the animal was placed in an appropriate transparent glass cylinder for 300 g animals (with 20 cm diameter and 30 cm height) that encourages vertical exploration [31] and video recorded for 3 min. Behavior data for only the first 20 trials were considered for comparison between groups and sessions to prevent habituation to the cylinder. The evaluation was performed in dark and at the same period of day, between 5 pm and 6 pm. Rats actively explore vertical surfaces by rearing up on their hindlimbs and exploring the surface with their forelimbs and vibrissae. Rats performed without any external motivation. Animals with unilateral brain damage display asymmetry in forelimb use during vertical exploration [31]. When assessing behavior in the cylinder, the number of independent wall placements observed for the right forelimb (contra), left forelimb (ipsi), and both forelimbs simultaneously (both) were recorded. The asymmetry score was calculated by a formula as described by Schaller [31], [(ipsi–contra) / (ipsi + contra + both)] x 100. The asymmetry score was converted to the symmetry score (100 –asymmetry score) for analysis [12,14–16,32–36].

### Statistical analysis

We used SPSS software version 24 [37] to analyze the behavior data. Generalized mixed models were used, and results were represented as estimated mean with a 95% CI. Multiple comparisons of repeat measures were performed for the motor assessment by actimeter and cylinder tests among the groups, and it was corrected by Bonferroni ad hoc test using the 1% level of significance. The data were represented as Box-Whister Plot with individual data points (dot plot).

## Results

### Thermocoagulation induction in somatosensory cortex vessels-results of thermal dissipation in brain tissue by hot probe

**Fig 2:**
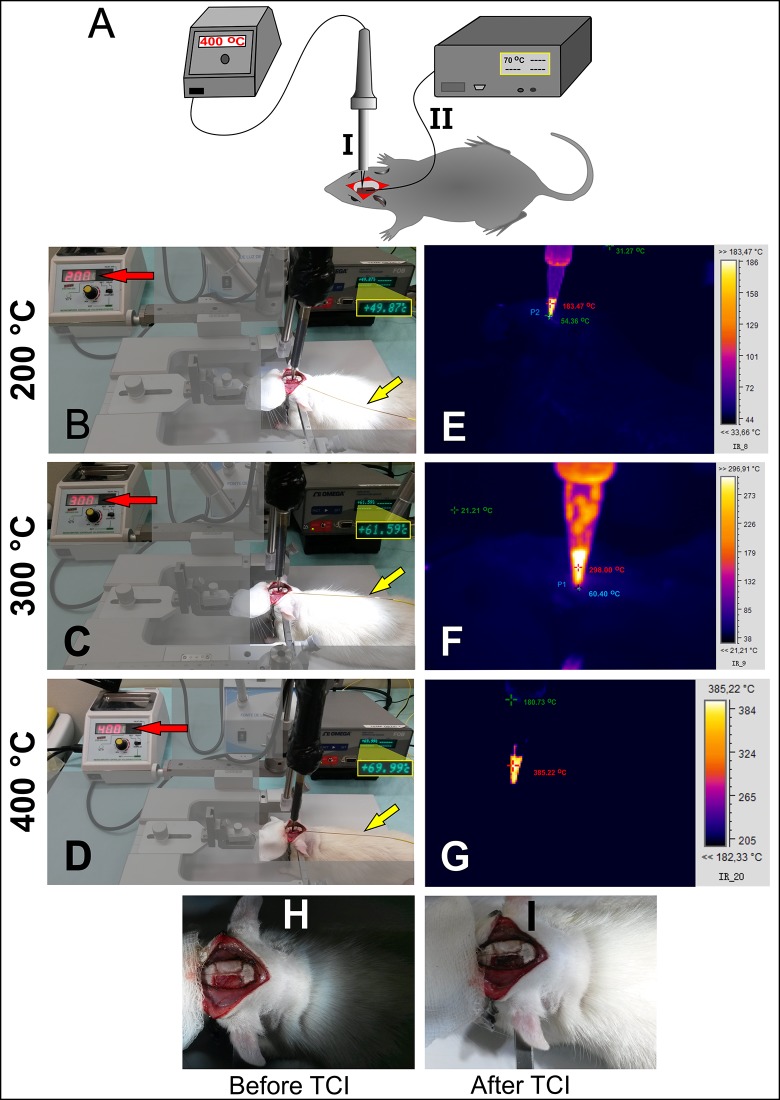
Ischemic lesion induction by thermocoagulation (Experiments 1 and 2). A-I: A hot probe with digital control of temperature, A-II: Fiber optic thermometer systems, B–D: Thermocoagulation induction (TCI) at 200°C, 300°C, and 400°C (red arrow) and thermal dissipation analysis in the brain tissue by using fiber optic at each temperature condition (yellow arrow) (*n* = 5 per group), E–G: Thermal dissipation analysis on the tissue brain through infrared camera at each temperature of induction, H: color of brain tissue before TCI (light red), and I: color brain tissue after TCI (dark red).

The ischemic lesions by TCI were evaluated with three different controlled temperatures using the hot probe (Fig 2A-I) and monitored by equipment that directly measured the thermal dissipation in the brain tissue (Fig 2A-II). On induction at 200°C, 49.80 ± 0.45°C was measured through fiber optics (Fig 2B), and the infrared camera detected average temperatures in the brain tissue at 54.36 ± 1.16°C and on the probe at 183.47 ± 1.12°C (Fig 2E). On induction at 300°C, the fiber optic registered 61.90 ± 0.52°C of thermal dissipation in the brain tissue (Fig 2C), and the infrared camera showed 60.40 ± 1.53°C on brain tissue and 298.0 0± 1.45°C on the probe (Fig 2F). On induction at 400°C, fiber optic showed 69.90 ± 0.57°C in the brain tissue (Fig 2D), but the spatial measured by the infrared camera registered only the probe temperature of 385.22 ± 2.10°C because change in the detection range precluded recording of temperatures below 182.33°C which was the lower limit of temperature for this range that could be measured by the infrared camera (Fig 2G). Detection after induction of lesion by thermocoagulation was possible because the tissue color changed from light red (Fig 2H) to dark red (Fig 2I). In the sham group, the fiber optic registered 35.50 ± 0.30°C of thermal dissipation in brain tissue, and the infrared camera measured 36.20±1.09°C in brain tissue.

### Real-time blood perfusion imaging analysis of an ischemic lesion

**Fig 3:**
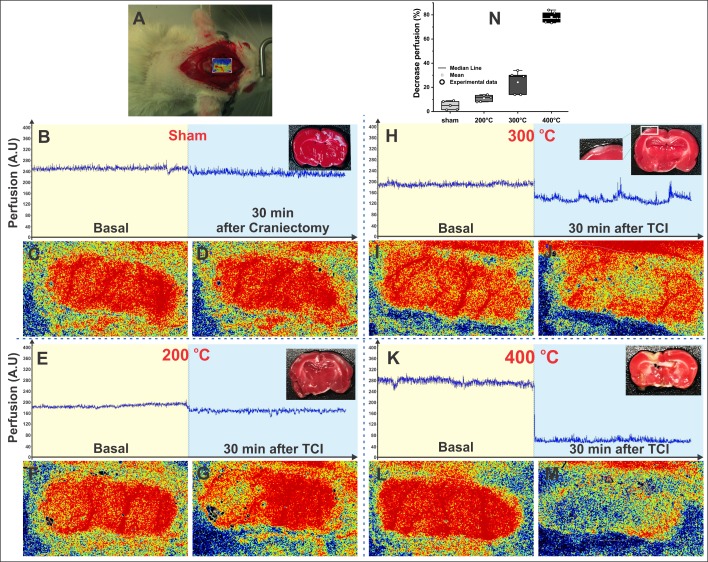
Blood perfusion image analysis of ischemic lesion area and TTC staining image (Experiment 1). A: Animal´s image during baseline perfusion analysis. B, E, H, and K: The blood perfusion values at basal and 30 min after craniectomy in the sham group (*n* = 5) and 30 min after TCI at 200°C, 300°C, and 400°C, respectively, in ischemic groups (*n* = 5 in each group), combined with coronal TTC-staining slices of animals 120 min after TCI in each condition (as seen in the upper left margin); C, F, I, and L: The blood perfusion basal images in each experimental condition; D: The blood perfusion image 30 min after craniectomy in sham condition; G, J, and M: The blood perfusion image 30 min after TCI at 200°C, 300°C, and 400°C, respectively; and N: Decrease in perfusion rate in each experimental condition, between the basal time (after craniectomy) and 30 min after TCI and the data were represented by the boxes that show the values of the 25th and 75th percentiles, the lines across the boxes represent the medians, and the whiskers extend to the highest and lowest values, using different shades of gray for each group.

Blood perfusion images of pial vessels in the left motor and sensorimotor brain regions before TCI served as the baseline measure. This basal perfusion was acquired after craniectomy (Fig 3A) in the experimental groups (Fig 3B, 3E, 3H and 3K).

At 30 min after TCI, blood perfusion was measured and compared with the basal measure. The blood perfusion rate showed a decrease of 5% ± 4% in the sham surgery group (Fig 3B–3D and 3N), a decrease of 11% ± 3% in the 200°C group (Fig 3E–3G and 3N), 24% ± 10% in the 300°C group (Fig 3H–3J and 3N), and 78% ± 5% in the 400°C group (Fig 3K–3M and 3N).

TTC staining complemented the blood perfusion analysis. The coronal slice of the brain in the upper left margin in Fig 3 shows the result. In Fig 3B, the sham group did not show the non-TTC-staining lesion. In Fig 3E, a light ischemic lesion in the pale area was confirmed by the non-TTC-stained area of the left sensorimotor cortex (cortical brain region) with a progressively increasing lesion for TCI at 300°C (Fig 3H) and extending to the corpus callosum and the subcortical brain region for TCI at 400°C (Fig 3K).

### Magnetic resonance imaging (MRI)

**Fig 4:**
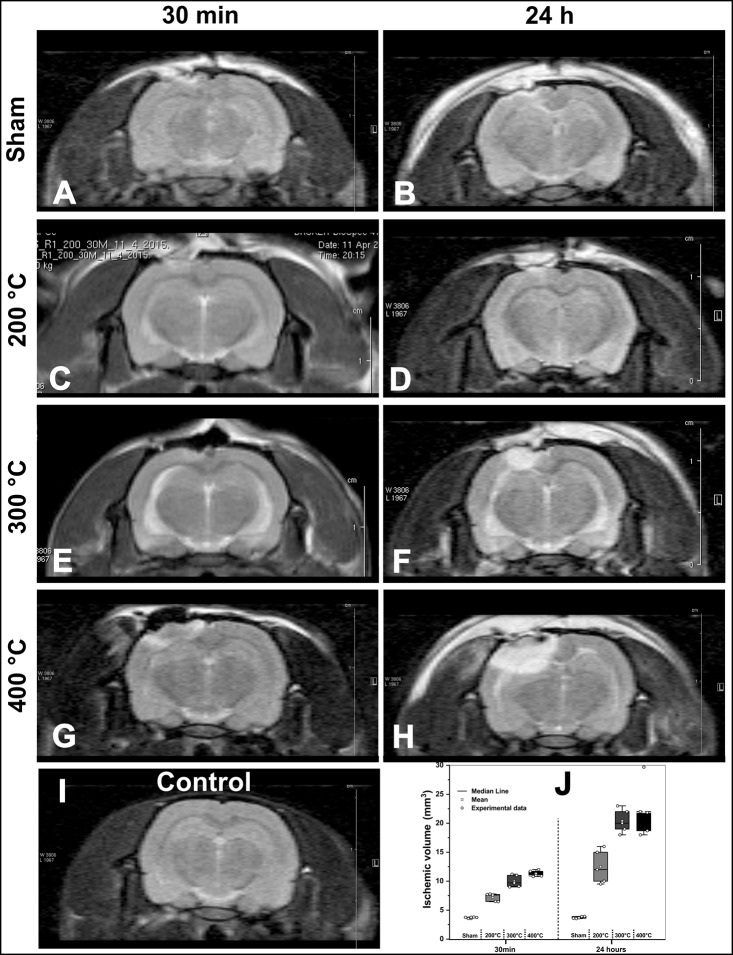
MRI of the ischemic model at 30 min and 24 h after TCI (Experiment 2). A–B: sham surgery; C–D: Ischemic with TCI at 200°C; E–F: Ischemic with TCI at 300°C; G–H: Ischemic with TCI 400°C, I: control animal’s MRI, and J: Volumetric results of each experimental condition by MRI were the data represented by the boxes that show the values of the 25th and 75th percentiles, the lines across the boxes represent the medians, and the whiskers extend to the highest and lowest values, using different shades of gray for each group (*n* = 25, five animals per group): sham surgery lesion, ischemic lesion at 200°C, ischemic lesion at 300°C, and ischemic lesion of 400°C.

MRI images of the sham group at 30 min and 24 h (Fig 4A and 4B) after TCI revealed a focal dural enhancement under the craniectomy area. MRI acquired at 30 min after TCI showed the left sensorimotor cortex having a discrete hypersignal for 200°C (Fig 4C) and 300°C (Fig 4E); for 400°C (Fig 4G), the hypersignal was clearly visible, extending to the corpus callosum, without causing any deformities.

The MRI at 24 h after TCI showed a progressive hypersignal with increase in temperature. In the coronal plane, the ischemic lesion image involved the cortical area of the left sensorimotor cortex at 200°C (Fig 4D), crossed the corpus callosum at 300°C (Fig 4F), and at 400°C, the ischemic lesion image (Fig 4H) represented around one-third of the left hemisphere, without any change in the alignment of the right hemisphere. Fig 1 represents the basal MRI in the control group.

In Fig 4J, the volumetric MRI of the sham surgery animals showed a similar brain volume throughout (3.70 ± 0.10mm^3^ and 3.70 ± 0.20 mm^3^). However, images of animals at different temperatures showed an increased lesional volume at 30 min and 24 h. For induction at 200°C, the ischemic lesion volume increased from 7.30 ± 0.70 mm^3^ to 12.70 ± 3.10 mm^3^; for 300°C, the volume increased from 9.90 ± 1.00 mm^3^ to 20.70 ± 2.10 mm^3^; and for 400°C, it increased from 11.30 ± 0.60 mm^3^ to 23.40 ± 5.70 m^3^.

### Behavior results in ischemic model

**Fig 5:**
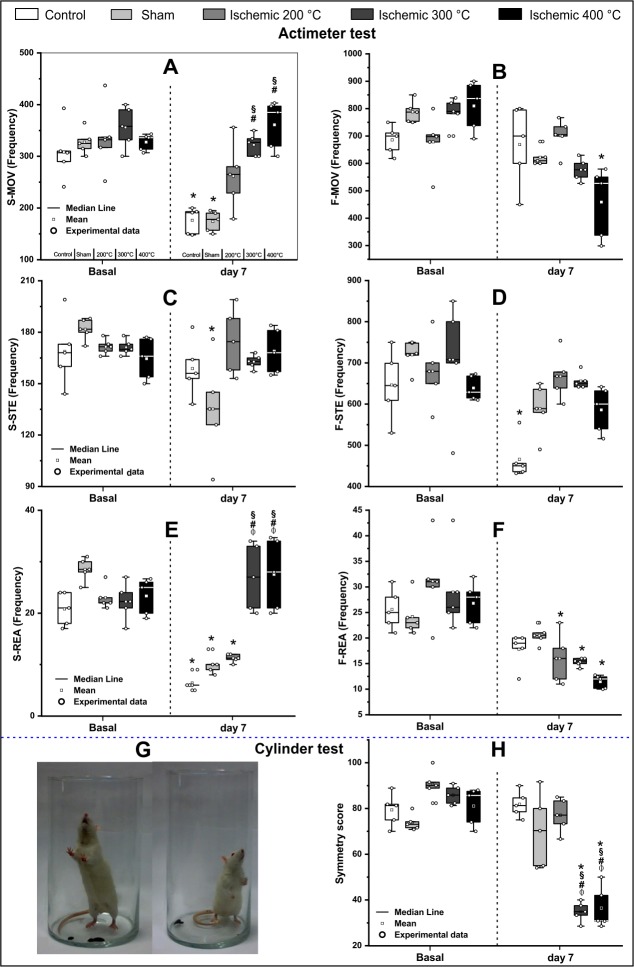
Motor behavior assessment using Actimeter and cylinder tests at basal and after 7 days of thermocoagulation induction (Experiment 2). In Actimeter test (A-F), A: Slow movement (horizontal activity), B: Fast movement (horizontal activity), C: Slow stereotyped, D: Fast stereotyped, E: Slow rearing movement (vertical activity), and F: Fast rearing movement (vertical activity). The cylinder test (G-H), G: Bilateral forelimb analyses-on the left, an animal with bilateral symmetry and on the right, an animal with asymmetry contralateral with the ischemic lesion, H: Symmetry score at basal time and seven days after induction. Both tests were performed in the five experimental groups (n = 5 per group): control, sham, ischemic with 200°C, ischemic with 300°C, and ischemic with 400°C, which were represented by the boxes that show the values of the 25th and 75th percentiles, the lines across the boxes represent the medians, and the whiskers extend to the highest and lowest values, using different shades of gray for each group. *: p<0.001 in comparison with base time, #: p<0.001 in comparison with control group, and §: p<0.001 in comparison with the sham group.

Spontaneous motor assessment with the actimeter test revealed slow movement and slow rearing parameters for the horizontal and vertical movement, respectively with significant results (*p* <0.001) for group and time effect as well as group and time of interaction. Fast movement, fast stereotyped, and fast rearing parameters showed significant results (*p* <0.001) only in time effect for comparison between basal time and the seventh day after induction (Table 1 and Fig 5A–5F).

**Table 1:** Effect time and group for Actimeter test.

Actimeter parameters	Group Effect		Time Effect		Group and Time Interaction	
	F (4;30)	p	F (1;30)	*P*	F (4;30)	p
**S-MOV**	9.805	<0.001	29.670	<0.001	6.410	<0.001
**F-MOV**	0.824	0.520	27.230	<0.001	5.701	0.002
**S-STE**	0.825	0.520	5.101	0.031	2.875	0.040
**F-STE**	3.763	0.013	14.654	<0.001	2.506	0.051
**S-REA**	22.033	<0.001	53.355	<0.001	18.187	<0.001
**F-REA**	0.676	0.614	77.841	<0.001	3.841	0.012

Generalization mixed model with first order auto regression structure for covariate. (n = 5 per group)

For a greater understanding of the differences in groups and for time comparison, we observed significant results (p <0.001) of time effect in the control group for S-MOV, F-STE, and S-REA parameters (Fig 5A, 5D and 5E); in the sham group for S-MOV, S-STE, and S-REA parameters (Fig 5A, 5C and 5E); in the 200°C group for S and F-REA parameters (Fig 5E and 5F); in the 300°C group for F-REA parameters (Fig 5F); and in the 400°C group for F-MOV and F-REA parameters (Fig 5B and 5F). The group effect was significant (p<0.001) for control and sham groups (as indicated with different symbols in Table 2 and Fig 5A and 5E) and 300°C and 400°C groups for S-MOV and S-REA. The last parameter was significant (p<0.001) when 300°C and 400°C groups were compared with the 200°C group.

**Table 2:** Estimated mean and 95% confidence interval of each group for spontaneous motor activity analysis.

Actimeter parameters	Time	Group				
		Control	Sham	200°C	300°C	400°C
**S-MOV**	0	308.2	328.3	334.5	355.5	327.0
		(266.8; 356.0)	(280.8; 383.7)	(286.6; 390.5)	(306.0; 413.1)	(273.0; 391.8)
	7	176.8	173.0	261.0	321.0	361.7
		(146.2; 213.9) *	(139.5; 214.5) *	(219.1; 311.0)	(274.1; 375.9) # **§**	(304.6; 429.5) # **§**
**F-MOV**	0	685.8	787.5	678.3	787.3	809.0
		(605.2; 777.2)	(691.2; 897.3)	(589.3; 780.6)	(690.9; 897.0)	(697.3; 938.6)
	7	669.0	622.5	700.3	576.8	458.7
		(589.4; 759.3)	(537.5; 720.9)	(609.8; 804.2)	(495.2; 671.7)	(376.5; 558.8) *
**S-STE**	0	168.8	181.8	171.5	171.5	165.7
		(153.2; 186.0)	(163.7; 201.8)	(154.0; 191.0)	(154.0; 191.0)	(146.0; 188.0)
	7	158.8	135.3*	174.5	162.5	168.7
		(143.7; 175.5)	(119.8; 152.7)	(156.8; 194.2)	(145.5; 181.5)	(148.8; 191.2)
**F-STE**	0	646.6	719.5	679.5	707.7	639.0
		(577.8; 723.6)	(638.6; 810.6)	(601.0; 768.2)	(627.6; 798.2)	(552.1; 739.5)
	7	465.8	589.0	667.7	656.0	586.0
		(408.0; 531.8) *	(516.3; 672.0)	(590.0; 755.7)	(579.0; 743.3)	(503.1; 682.6)
**S-REA**	0	20.8	28.5	23.0	22.3	23.3
		(17.6; 24.6)	(24.2; 33.5)	(19.2; 27.5)	(18.5; 26.7)	(19.0; 28.7)
	7	6.4	10.0	11.3	27.0	27.3
		(4.7; 8.7) *	(7.6; 13.1) *	(8.7; 14.5) *	(22.9; 31.9) # **§****ϕ**	(22.6; 33.1) # **§****ϕ**
**F-REA**	0	25.6	24.3	31.0	29.0	27.3
		(21.1; 31.0)	(19.4; 30.3)	(25.5; 37.7)	(23.7; 35.5)	(21.5; 34.8)
	7	17.6	20.5	16.0	15.5	11.3
		(13.9; 22.2)	(16.1; 26.1)	(12.2; 21.0) *	(11.8; 20.4) *	(7.8; 16.5) *

Abbreviations: S-MOV–slow movement, F-MOV–fast movement, S-STE–slow stereotypic, F-STE–fast stereotypic, S-REA–slow rearing, and F-REA–fast rearing. Data are described by estimated mean (95% confidence interval). (n = 5 per group)

Multiple comparison analysis corrected by Bonferroni for time and groups

*: p<0.001 in comparison with base time

#: p<0.001 in comparison with control group

§: p<0.001 in comparison with sham group

ϕ: P<0.001 in comparison with the 200°C group

The rodent´s spontaneous forelimb use as obtained from cylinder test measures for vertical-lateral functional exploration (Fig 5G) showed a reduction in the symmetry score for all groups at basal time; seven days after induction, the cylinder results were significant (p<0.001) for group and time effect as well as for group and time interaction (Table 3 and Fig 5H).

**Table 3:** Effect time and group for symmetric score by cylinder test.

Effect		Symmetry Score (%)
Group	F (g.l.: 4, 30)	13,583
	p	<0.001
Time	F (g.l.: 1, 30)	53,891
	p	<0.001
Group x time interaction	F (g.l.: 4, 30)	13,442
	p	<0.001

Generalization mixed model with first-order auto regression structure for covariate. (n = 5 per group)

In Table 4, we observe significant results (p <0.001) for time effect in the 300°C and 400°C groups and group effect in the comparison between 300°C and 400°C groups with control, sham, and 200°C groups, respectively (Fig 5H).

**Table 4:** Estimated mean for symmetric score by cylinder test.

Time	Group				
	Control	Sham	200°C	300°C	400°C
0	79.4(71.2; 87.6)	73.9(64.7; 83.1)	90.5(81.4; 99.7)	85.9(76.7; 95.0)	81.1(70.5; 91.7)
7	81.9(73.7; 90.1)	70.3(61.1; 79.5)	77.1(67.9; 86.3)	34.9(25.7; 44.0) * # **§****ϕ**	36.4(25.9; 47.0) * # **§****ϕ**

Data described by estimated mean (95% confidence interval). (n = 5 per group)

Multiple-comparison analysis corrected by Bonferroni for time and groups

*: p<0.001 in comparison with base time

#: p<0.001 in comparison with control group

§: p<0.001 in comparison with sham group

ϕ: P<0.001 in comparison with the 200°C group

No animals were excluded or died during the experiments which proves the low mortality of the thermocoagulation method [5,6].

## Discussion

This study assessed the functional outcome and histological damage for thermocoagulation at 400°C as being the most effective. However, for lesion induction at 300°C, thermal dissipation in the brain is lower than the level effective to induce an adequate thermocoagulation ischemic lesion. At 300°C, the temperature decreases by only 24% of the local perfusion below 70°C, but significant results were obtained in behavior function in both tests. In addition, an ischemic lesion was detected by TTC and MRI involving the area of interest in the ipsilateral sensorimotor cortex.

For improved reproducibility of stroke induction, this study first analyzed the thermal dissipation parameter by two precise temperature survey systems in the ischemic lesion process, whose range did not detect the lower value during the lesion induction at a high temperature of 400°C, but the temperature measures of the probe were identical for all temperatures of induction. This parameter is not used often in preclinical experiments [38,39], but the increased interest in clinical studies about brain heat after deep brain stimulation for the treatment of medically refractory movement disorders as well as other neurological and psychiatric conditions [40] go back to the preclinical studies about the thermal impact of implants on brain tissue. Nonose et al., (2017) [13] showed metabolic changes (glutamate uptake and lactate oxidation) at near and far cortical areas after increased thermal induction. Therefore, the heat transfer control is a pivotal aspect in this ischemic model.

Analysis of heat transfer process by external sources as is the case of the thermocoagulation ischemic brain model can be understood by Pennes bio-heat equation based on the heat diffusion equation [41]. From this equation, it is possible to understand that blood coagulation occurs around 70°C as reported in the perfusion experiment [42], but this value depends on several factors such as vessel size, duration of external heat source induction, and arterial or venous vessel pressure at each brain point, among others [43–45]. In this study, the thermal dissipation on tissue brain achieved was 69.9°C only with 400°C of TCI for blood coagulation.

The blood perfusion measure after TCI showed decreasing perfusion with increasing temperature of induction. Time of application was identical and the real-time analysis by laser speckle contrast imaging confirmed the vessel occlusion non-invasively. This system for analysis of regional cerebral blood flow (rCBF) was also reported in the stroke model by intraluminal occlusion of the left middle cerebral artery for 60 min with an average 80 percent decreased perfusion compared to the baseline measure [24]. Yang [24] and Li [25] used the same model with 60 min of occlusion and reported exclusion criteria for rCBF reduction inferior of 75 percent on laser speckle imaging, considering vascular occlusion for value above this limit [25]. With the same system of perfusion analysis, we found on average 78 percent of rCBF reduction only in the 400°C induction group, but with 30 min of induction, which is half the time of induction mentioned in the above studies [24,25]. Considering the influence of exposure time of heating, with double the time, our group with 300°C induction might have achieved the appropriate percentage to confirm vascular occlusion [24,25]. Nonose et al., (2017) [13] showed increase in late-onset glial reactivity after rCBF reduction. This neurobiological event has the potential for translational research. In the penumbra area human brain, late-onset glial reactivity without window time of rTPa (recombinant tissue plasminogen activator) administration has been observed (Garbuzova-Davis et al., 2017) [46] as well as associated with an increased diffusion signal in the MRI in this area.

This study additionally used conventional analysis to confirm ischemic lesion, the MRI (basal, 30 min, and 24 h after ischemic induction) and TTC staining (basal and after 2 hours post-ischemic induction).

MRI represents an adequate technique to analyze the ischemic lesion especially for the results of acute brain edema due to the lesion [47]. After ischemic lesion induction, activation of astrocytes induces an expression of aquaporin-4 that results in water congestion in the lesion area and hypersignal in the MRI [48]. An MRI hypersignal was observed 30 min after TCI with 200°C (Fig 4C), 300°C (Fig 4E), and 400°C (Fig 4G), borne out by studies of the stroke model that used MRI in early stage to show hypersignal in ischemic brain lesions [49, 50].

The MRI images 24 h after ischemic lesion induction had a considerable increase measured by morphometric analysis compared with MRI after 30 min (Fig 4J); these findings were reported in studies of ischemic lesions that compared temporal evolution of ischemic lesions [51]. The small brain lesion caused by sham surgery did not change the brain volume in this group and did not cover deep cortical areas (Fig 4B). The ischemic lesion was detected and measured from the thermal dissipation in the tissue by induction as reported in studies that used the same focal ischemic cerebral model [12–15].

In this study, TTC staining was used for histological analyses of the lesion area 2 h after TCI (Fig 3) and confirmed the extension of the lesion into the cortical and subcortical regions. This sensitivity analysis of TTC has been described in previous studies with the same ischemic model [15,16]. The ischemic lesion by the thermocoagulation model compromised all cortical layers around the vessel coagulated at five to seven days after induction detectable by TTC and histological analysis [11,14,16], keeping the integrity of the medial cortex, corpus callosum, striatum, and subventricular zone due to blood supply by anterior and middle cerebral arteries [19]. The histological analysis corroborated MRI analysis as in Hansel’s [14] study.

Besides histological and MRI evidence of thermal induction ischemia lesion, this study used observational tests to assess motor behavior. The TCI study by Giraldi-Guimarães et al., (2009) showed significant decrease in behavioral and histological aspects to confirm the ischemic lesion, but did not show any response to the temperature of the probe [16]. In our study, the 300°C and 400°C experimental groups showed the same pattern of decrease in fast movements (actimeter test) and percentage of symmetric score (cylinder test) comparing with other groups and over time, but the slow movements had similar performance over time with a slight increase in slow vertical movement and a significant difference in comparison with control and sham groups at seven days after TCI.

Studies with stroke model that analyzed the vertical and horizontal movements after brain lesion using open field test reported a reduction of locomotor activity at 3, 7, and 14 days after ischemic induction by comparing all experimental groups to basal time, but in ischemic groups the significant results indicated motor impairment deficit with habituation effect [52–54].

Nonetheless, the opposite direction of movement changes after ischemic induction was reported with an increased number of vertical and horizontal movements in open field test between ischemic and sham groups at seven days after ischemic induction. This behavioral change was justified by a loss of habituation in the ischemic group [55]. In our results, the 300°C and 400°C groups showed this variation in slow vertical and horizontal movement parameter (Actimeter) in comparison with control and sham groups. Loss of habituation may be the justification for this result, but no other study found that analysis of the pattern of movement to explain this fact in slow movement and not in fast movement.

The significant change in symmetric score of the cylinder test was also reported in other studies with the permanent focal cerebral ischemic injury by thermocoagulation in the left motor and sensorimotor cortices, but these studies did not specify the temperature used and, only reported that the temperature of the was near 400°C [11,12,14,16–18]. The symmetric score decreased significantly in animals that underwent TCI with 300°C and 400°C in comparison with other groups, at seven days after of ischemic induction.

The motor deficit observed in these animals represents the cortical sensory motor lesion in the rat, located in the frontal cortex, rostrally in relation to the parietal cortex. This area has a dense projection to the striatum, sub-thalamic nucleus, cerebellum, and other sub-cortical structures that play an important role in the motor control [56], and the sensitivity of the cylinder test is used to detect the sensorimotor lesion [17].

Findings observed in motor function in both tests confirmed the motor deficit caused by TCI as at 300°C or above, although the 300°C group did not show reduced perfusion to consider as complete vascular occlusion according to results from other groups [24,25].

The thermocoagulation represents a permanent and efficient focal cerebral ischemic model; however, this model needs to be standardized for certain details such as probe temperature, induction time, and precise parameters to consider the complete ischemic lesion; none of this information has been reported in previous studies [11,14,17–19,57]. In addition, despite many studies showing the brain lesion and the deficits caused by TCI, only a few have reported an indicator of complete ischemic injury based on color change from light red to dark red after thermocoagulation [12,14,17,19]. Also, this study had some restrictions, especially concerning the MRI equipment for the acquisition of the perfusion sequence, as well as the data of TTC and MRI analysis, which were acquired with a span of 22 hours between them, therefore not permitting the analysis of co-relation with the size of the lesion.

Finally, we assume that this study played a role in understanding some aspects of the focal thermocoagulation ischemic brain model unclear in previous studies that might contribute to future studies. We showed that the best condition for the permanent focal thermocoagulation ischemic brain model induction was 400°C of temperature for 30 min, resulting in complete brain vascular occlusion with more than 75 percent of blood perfusion reduction and a thermal dissipation in brain tissue around 69.90°C, as well as effective motor behavior deficits and relevant brain ischemic lesion detected by MRI and TTC images.
